# Sublingual nitroglycerin for early blood pressure control in hypertensive emergencies: observations from an emergency department clinical audit in Sri Lanka

**DOI:** 10.1186/s13104-018-3460-0

**Published:** 2018-06-05

**Authors:** Nadarajah Prasanna, Harsha A. Dissanayake, Godwin R. Constantine

**Affiliations:** 1 Emergency Medicine, Post Graduate Institute of Medicine, University of Colombo, Colombo, Sri Lanka; 2 University Medical Unit, National Hospital of Sri Lanka, Colombo, Sri Lanka; 3 Department of Clinical Medicine, University of Colombo, Colombo, Sri Lanka

**Keywords:** Hypertensive emergency, Sublingual nitroglycerin, Blood pressure control

## Abstract

**Objective:**

Hypertensive emergencies are potentially life threatening and require prompt blood pressure control with intravenous agents. Preparation of intravenous infusions is time consuming. Usefulness of sublingual nitroglycerin in this setting is not known. We aimed to assess the benefit of sublingual nitroglycerin as a bridge to IV therapy. In a clinical audit in an emergency department, patients presenting with hypertensive emergencies requiring intravenous nitroglycerin were administered single spray of sublingual nitroglycerin awaiting commencement of intravenous infusion. Blood pressure was monitored every 5 min to observe the degree and speed of reduction.

**Results:**

Thirty-seven patients met the selection criteria. Mean age was 65.8 years (SD 7.04), and 29 were males (88.4%). Mean values of systolic, diastolic and mean blood pressures on admission were 217, 137, 163 mmHg. At 5 and 10 min after sublingual nitroglycerin, mean reduction of mean arterial blood pressure by 12.3 and 16.3% was achieved. Only 2 patients (5.4%) showed an overcorrection of blood pressure. Minimum of 15 min were required to set up a nitroglycerin intravenous infusion. Sublingual nitroglycerin spray allows rapid blood pressure control in hypertensive emergencies and is a useful bridge during the time to prepare intravenous infusion.

## Introduction

Hypertensive emergency is an acute elevation of blood pressure (180/120 mmHg) associated with end organ damage [1]. In Sri Lanka, hypertensive diseases were the twelfth leading cause for in-hospital deaths during 2015 (3.4 deaths per 100,000 population) while deaths due to Ischemic Heart Diseases are the leading cause (29.7 deaths per 100,000) [2].

Nitroglycerin is a potent venodilator, showing arterial dilation only at very high doses. The use of sublingual, topical or IV nitroglycerin infusion is recommended as a first line agent in blood pressure control in acute hypertensive pulmonary oedema and in acute coronary syndrome due to its favourable effect in reducing preload and cardiac output [3]. It is useful as second line agent in hypertensive emergencies due to acute sympathetic crisis (as in cocaine, amphetamines, monoamine oxidase inhibitor toxicities, Irukandji Syndrome) [3]. The recommended mean arterial pressure (MAP) reduction in these conditions is up to 25% for the first 24 h, and over-reduction might cause end organ ischemia and more harm [3].

Sublingual nitroglycerin is widely used for its antianginal properties. It is unlikely to be useful for definitive management of hypertensive emergencies or urgencies due to short duration of action and variable absorption. However, its convenient use, ease of administration and rapid onset of action make it an ideal agent for rapid blood pressure control until definitive therapy is commenced in the emergency department or in pre-hospital setting. There are no studies describing this utility of sublingual nitroglycerin. We analysed data from a clinical audit to assess the effectiveness of sublingual nitroglycerin spray, as a bridging option during the inevitable delay in setting up IV nitroglycerin.

## Main text

### Methods

#### Design and setting

A clinical audit was conducted at the emergency department of a tertiary care centre in Sri Lanka from August, 2017 to December, 2017. Patients presenting with hypertensive emergencies who required intravenous nitroglycerin administration were selected by convenient sampling.

#### Selection of participants

Inclusion criteria were severe hypertension (defined as systolic blood pressure 180 mmHg or more and/or diastolic blood pressure 120 mmHg or more) with evidence of end organ dysfunction. Presence of clinical features of heart failure (dyspnoea, orthopnoea, bibasal crackles, hypoxia SpO2 < 94% in the absence of chronic lung disease), or angina with electrocardiographic changes suggestive of ischemia or neurological symptoms and/or signs (severe headache, visual blurring, altered conscious level, papilloedema) were considered as evidence for end organ dysfunction. Suspected or confirmed aortic dissection, intracranial haemorrhage or stroke were excluded from the study as their blood pressure goals and pharmacological preferences are different. All participants or next of kin (where patients’ capacity was limited due to the acute illness) provided informed consent prior to selection.

#### Observations

Upon diagnosis of hypertensive emergency that require IV nitroglycerin infusion, patients were administered single puff of nitroglycerin spray sublingually (0.4 mg). Blood pressure was monitored every 5 min by automated monitoring machines using arm blood pressure cuff and was confirmed with manual blood pressure measurement with a mercury sphygmomanometer and systolic, diastolic and mean arterial blood pressures (SBP, DBP and MAP respectively) were recorded. Time taken to prepare the IV nitroglycerin infusion was noted. Other regular medical management was continued unchanged according to unit protocols.

### Results

Thirty seven patients met the selection criteria for enrolment. Mean age was 65.6 years and 29 were males (88.4%). Their clinical presentations are summarized in Table 1.

**Table 1:** Clinical presentations of hypertensive emergencies (N = 37)

Presentation	Number of patients	Percentage (%)
Pulmonary oedema	16	43.2
Acute coronary syndrome	7	18.9
Neurological symptoms/signs (excluding stroke)	14	37.9

Table 2 shows the blood pressure on admission and the reductions in blood pressure 5 and 10 min after sublingual nitroglycerin. Rapid blood pressure reduction was achieved with single puff of sublingual nitroglycerin spray.

**Table 2:** Blood pressure on admission and after treatment with sublingual nitroglycerin

	On admission	5 min after spray		10 min after spray	
		Mean	Reduction from baseline (%)	Mean	Reduction from baseline (%)
Systolic BP	217.0 (14.7)	197.8 (13.3)	8.7	188.5 (9.6)	13.1
Diastolic BP	136.2 (9.6)	115.5 (12.6)	15.1	110.3 (9.9)	19.0
Mean BP	163.2 (8.1)	143.0 (9.9)	12.3	136.4 (7.8)	16.3

Blood pressure values are mean (SD). *BP* blood pressure

Only one patient had over-reduction of MAP more than 25% at 5 min (28%), which improved to 22% at 10 min, while another patient who had 18% reduction at 5 min had further reduced MAP by 29.5% at 10 min.

Minimum delay from decision to commence IV nitroglycerin to commencement of therapy was 15 min (mean 27 min) and was due to delays in gaining IV access and preparation and setting up of the solution for infusion.

### Discussion

Rapid and controlled reduction of blood pressure is the main aim in management of hypertensive emergencies. Intravenous administration of antihypertensives are required. However, setting up an intravenous infusion consumes time, during which patient remains symptomatic and may even deteriorate. We observed that mean time taken for commencement of IV infusion from the time of deciding to treat is 27 min. Fastest time achieved was 15 min.

Therefore it is important to have an alternative strategy which can be rapidly administered and could produce rapid effect. It is not critical for it to be highly efficacious or long lasting as its role is to bridge the time until definitive IV preparation is ready to use. Nitroglycerin sublingual spray has the advantages of being easy to administer (without a need for IV access) and having rapid absorption and onset of action. Therapeutic utility of nitroglycerin has been questioned at times due to its venodilatory effects, potential to reduce cardiac output and cause reflex tachycardia [4]. However one recent study demonstrated effective blood pressure reduction with sublingual nitroglycerin in hypertensive urgencies [5]. Yet it is not known whether sublingual nitroglycerin could achieve clinically relevant blood pressure reduction for a brief duration in a hypertensive emergency. Our analysis was aimed to answer this question.

Findings of this clinical audit demonstrate that sublingual nitroglycerin is efficacious, rapid in onset and achieved desired blood pressure control. This is in keeping with its known pharmacokinetic properties, being rapidly absorbed through mucous membrane of oral cavity. Only one patient experience sustained over correction of blood pressure, exceeding 25% reduction of MAP.

These findings have two important implications. Firstly, we suggest that sublingual nitroglycerin spray can be a useful adjunct in the management of a hypertensive emergency until parental preparation is prepared, thus preventing complications due to unavoidable delays. Secondly, this also indicates the potential usefulness of sublingual nitroglycerin spray in out-of-hospital setting for paramedical services in better management of hypertensive emergencies.

Therefore we conclude that sublingual nitroglycerin spray is effective in reducing blood pressure in a hypertensive emergency, until IV preparation is made available. We suggest that potential use of sublingual nitroglycerin in hypertensive emergencies should be studied further, in a larger population, with a control group with prospective follow up to determine final outcome.

## Limitations

There are few limitations in our study. Firstly, it is limited by small sample size. Secondly, effect of nitroglycerin was not compared against a control or a placebo. Thirdly, effect of early correction of high blood pressure on mortality, duration of hospital stay, need for other antihypertensives or morbidity were not assessed. Fourthly we did not observe any adverse effects of therapy probably due to the good safety profile of this medication and also due to the small sample size that was not adequate to determine more rare adverse events.

## Abbreviations

BP: blood pressure
DBP: diastolic blood pressure
IV: intravenous
MAP: mean arterial pressure
SBP: systolic blood pressure
